# RepSeq Data Representativeness and Robustness Assessment by Shannon Entropy

**DOI:** 10.3389/fimmu.2018.01038

**Published:** 2018-05-15

**Authors:** Wahiba Chaara, Ariadna Gonzalez-Tort, Laura-Maria Florez, David Klatzmann, Encarnita Mariotti-Ferrandiz, Adrien Six

**Affiliations:** ^1^Sorbonne Université, INSERM, UMR_S 959, Immunology-Immunopathology-Immunotherapy (i3), Paris, France; ^2^AP-HP, Hôpital Pitié-Salpêtrière, Biotherapy (CIC-BTi) and Inflammation-Immunopathology-Biotherapy Department (i2B), Paris, France

**Keywords:** TCR repertoire, diversity, sampling, normalization, bioinformatics

## Abstract

High-throughput sequencing (HTS) has the potential to decipher the diversity of T cell repertoires and their dynamics during immune responses. Applied to T cell subsets such as T effector and T regulatory cells, it should help identify novel biomarkers of diseases. However, given the extreme diversity of TCR repertoires, understanding how the sequencing conditions, including cell numbers, biological and technical sampling and sequencing depth, impact the experimental outcome is critical to proper use of these data. Here, we assessed the representativeness and robustness of TCR repertoire diversity assessment according to experimental conditions. By comparative analyses of experimental datasets and computer simulations, we found that (i) for small samples, the number of clonotypes recovered is often higher than the number of cells per sample, even after removing the singletons; (ii) high-sequencing depth for small samples alters the clonotype distributions, which can be corrected by filtering the datasets using Shannon entropy as a threshold; and (iii) a single sequencing run at high depth does not ensure a good coverage of the clonotype richness in highly polyclonal populations, which can be better covered using multiple sequencing. Altogether, our results warrant better understanding and awareness of the limitation of TCR diversity analyses by HTS and justify the development of novel computational tools for improved modeling of the highly complex nature of TCR repertoires.

## Introduction

Understanding the specificity of T cells involved in immune responses is of utmost importance in many fields of immunology. T cells are characterized by the expression a unique T cell receptor (TR), which is clonally generated by somatic rearrangement of the V, D, and J genes belonging to the TR genomic locus during thymic T cell differentiation (1). This process leads to the generation of a huge diversity of TR, defining a repertoire of antigen recognition, the hallmark of the adaptive immune response. Immunoscope analysis (also called CDR3 spectratyping) has long been the standard technique for TR repertoire analyses (2). Although immunoscope analysis has been very useful, it misses the key parameters of TR diversity, which include nucleotide sequence, codon usage, and amino acid composition. High-throughput sequencing (HTS) of the adaptive immune receptor rearrangements (RepSeq) expressed in a lymphocyte population now overcomes previous limitations, providing a thorough and multifaceted measure of diversity (3). Several studies have already highlighted the feasibility of HTS for the analysis of TR repertoire diversity in various immune contexts (4–17). However, while the amount of information and the depth of analysis provided by this technique are unprecedented, the representativeness and robustness of the data obtained remain to be established.

First of all, although not addressed in this study, the type of starting material (DNA/RNA) as well as the molecular biology method used to prepare a TR/IG template may impact the resulting diversity observed. Indeed, 5’RACE-PCR and multiplex-PCR, the two major methodologies used for TR/IG template amplification, can both introduce biases. Multiplex-PCR is mainly sensitive to primer competition and does not allow new variant identification, while 5’RACE-PCR will be sensitive to transcript integrity and length (18). An additional issue is the quantification of the species. Unique molecular identifiers have been proposed as a molecular method to trace the origin of identical species, thus distinguishing species arising from different cells or from PCR amplifications (19–22). A comparative study considering UMI on TR sequences obtained by 5’RACE-PCR or not suggested fewer intersample variations in quantification of unique TRB clonotypes based on sequences identified with UMI in comparison with randomly selected sequences (23, 24). However, amplification and sequencing errors in those highly variable short oligonucleotides can still occur and be difficult to assess and correct. In addition, UMI can be used only in 5’RACE-PCR methods. Therefore, not all the commercially available protocols include UMI and tools to handle them may need further improvement (25).

RepSeq is a numbers game (26) particularly dependent on sequencing depth and therefore on sampling. When monitoring T cell leukemia or highly expanded antigen-specific TCRs following an infection, the sampling and depth of sequencing might not be critical parameters. But things are different when studying TR repertoire diversity in physiological conditions, when describing the basics of immune repertoire generation and selection or in immune contexts where subtle or qualitative modifications may be involved in the pathophysiological outcome, such as in complex infectious diseases (27–29), autoimmune disorders (13, 30–35), and transplantation follow-up (36–38). However, RepSeq necessarily implies sampling: (i) only a fraction of the cells from peripheral blood or an organ (or a fragment of that organ in humans) is harvested; (ii) only a fraction of the RNA/DNA extracted from these cells is used for sample preparation; and finally, (iii) only a fraction of the library is used for a sequencing run. These different levels of experimental sampling are likely to affect the observed diversity.

This is a genuine issue described in ecology studies, as “the absence of observation of a species can be either real or the effect of a subsampling” (39). Previous studies showed that the number of clonotypes observed is positively correlated with sampling size (30, 40, 41). This is important, as studies performed in humans are mostly based on peripheral blood, a compartment that represents only around 2% of the total T lymphocyte population. Warren et al. (42) compared TR repertoires from two blood samples from the same individual and found a limited number of shared clonotypes (~10%). They concluded that a considerable proportion of the peripheral blood TR repertoire is unseen when observed randomly (42, 43).

The depth of the sequencing is another confounding factor for TR repertoire diversity studies, since an insufficient number of sequences produced would not adequately assess the molecular diversity of the sample analyzed. To ensure the statistical representativeness of the data produced with regards to the population of interest, two rules should be considered (44): (i) the number of sequences produced must be at least equivalent to the clonal richness of the population of interest and (ii) the rarer a clone, the greater the sequencing depth needed to detect it. Therefore, the RepSeq strategy must be adapted to the nature of the samples and the biological questions investigated (45).

While most studies seek to assess the similarity between the TR repertoires of several samples, without any knowledge of what level of similarity can be observed at best, it seems crucial to determine the limits of this approach in order to be able to interpret the data properly. In this study, we first investigated the impact of the depth of sequencing, in relation to the size of the population analyzed, on the observed TR repertoire diversity. We found that a small sample size is negatively affected by a too high, yet average in common practice, sequencing depth, and proposed an analytical approach to recover the “true” repertoire diversity. We then questioned the representativeness of a single RepSeq experiment by multiple sequencing of the same sample and demonstrated that performing a single sequencing run, even at high depth of sequencing, does not allow exhaustive observation of the existing clones in a polyclonal population. Finally, we addressed these experimental biases by computational simulation on RepSeq data reflecting several levels of clonality and sequencing depth, to have a better assessment of the robustness of the experimental observations.

## Materials and Methods

### Mice

Eight- to twelve-week-old female Balb/C Foxp3-GFP (C.129 × 1-Foxp3tm3Tch/J) and 24- to 26-week-old male C57Bl/6 Foxp3-GFP mice, both expressing the green fluorescent protein (GFP) under the promoter of Foxp3 gene, were, respectively, provided by V. Kuchroo, Brigham and Women’s Hospital, Boston, MA, USA and B. Malissen of the Centre d’Immunologie de Marseille Luminy (France). All animals were maintained in the Sorbonne Université Centre d’Expérimentation Fonctionnelle animal facility under specific pathogen-free conditions in agreement with current European legislation on animal care, housing, and scientific experimentation (agreement number A751315). All procedures were approved by the local animal ethics committee.

### Cell Preparation

Fresh total cells from spleen were isolated in PBS1X-3% fetal calf serum and stained for 20 min at 4°C with anti-Ter-119-biotin, anti-CD11c-biotin, and anti-B220-biotin antibodies followed by labeling with anti-biotin magnetic beads (Miltenyi Biotec) for 15 min at 4°C. B cells and erythrocytes were depleted on an AutoMACS separator (Miltenyi Biotec) following the manufacturer’s procedure. Enriched T cells were stained with anti-CD3 APC, anti-CD4 Horizon V500, anti-CD8 Alexa 700, anti-CD44 PE, and anti-CD62L efluor 450. 6.10^5^ CD3^+^CD4^+^GFP^−^ Teff cells were sorted on a BD FACSAria II (BD Biosciences, San Jose, CA, USA) with a purity >99%. Sorted cells were stored in Trizol (Invitrogen) or RNAAquous (Ambion, Inc./Life Technologies, Grand Island, NY, USA) lysis buffer.

### TR Library Preparation

RNA was extracted following the manufacturer’s recommendations and cDNA synthesis was performed with the Qiagen OneStep RT-PCR kit (Qiagen Inc., Valencia, CA, USA) and mouse T cell beta receptor primers provided with the mouse TRB iR-Profile Kit (iRepertoire Inc., Huntsville, AL, USA). cDNA was amplified by two rounds of PCR according to the manufacturer’s recommendations. The TRB library was sequenced using Illumina on a MiSeqv2 kit.

### RepSeq Data Processing

#### Data Annotation

The RepSeq fastq files were demultiplexed by iRepertoire Inc. and then annotated using clonotypeR (46) to identify high-quality productive and non-ambiguous TRB sequences. Clonotypes were defined as unique combinations of TRBV-CDR3-TRBJ segments.

#### Sequencing Error Correction

Annotated sequences were clustered per TRBV-TRBJ combination and similar clonotypes collapsed as follows: within each TRBV-TRBJ cluster, the clonotypes observed once (singletons) were separated from the others to constitute two groups. A Levenshtein distance was then calculated between the CDR3 peptide sequences of each clonotype of the two groups. The Levenshtein distance (lev) is a string metric measuring the minimum number of single-character edits (insertions, deletions, or substitutions) required to change one sequence into another (47).

When comparing the CDR3 peptide sequences of singleton with that of a “non-singleton” sequences, if lev_seq1,seq2_ = 1, their respective nucleotide sequences are then compared. If the two corresponding nucleotide sequences are also distant by 1, the singleton is considered as erroneous and considered as the “non-singleton” clonotype.

#### Dataset Normalization

Using the function *rrarefy* from the Vegan R package (48), randomly rarefied datasets were generated to given sample sizes. Random rarefaction was done without replacement.

### Diversity Profiles

Rényi entropy is a generalization of Shannon entropy, initially developed for information theory. We applied this mathematical function to clonotype frequencies to assess their diversity within each dataset. Rényi entropy is function of a parameter α, a strictly positive real number that differs from 1 and allows the definition of a family of diversity metrics spanning from (i) the species richness (α = 0), which corresponds to the number of clonotypes regardless of their abundance, to (ii) the clonal dominance (α → + ∞), corresponding to the frequency of the most predominant clonotype. For α = 1, the Shannon diversity index is computed. The exponential of the Rényi entropy defines a generalized class of diversity indices called Hill diversities, which can be interpreted as the effective number of clonotypes in the datasets (49) and thereby is used to build a diversity profile.

### RepSeq Simulation Algorithm

- 2·10^6^ clonotype library construction with the tcR package
  Based on the estimated total number of clonotypes in a mouse, a 2·10^6^ TRB CDR3 library was generated with the tcR package following the probability rules of V(D)J rearrangement established in Murugan et al. (50):
  $$\begin{array}{l} \Omega =\{\omega_{1};\omega_{2};\ldots \omega_{\Lambda }\},\text{ with }\Lambda =2\cdot 10^{6}\\ \forall i,\omega_{i}\;\text{is a clonotype generated by the tcR package}\\ \forall i,j,\omega_{i}\neq \omega_{j} \end{array}$$
- Construction of 6·10^5^ sequence datasets following particular Zipf distributions

Based on the demonstration by Greiff et al. (41) that clonotype frequencies determined from RepSeq datasets generally follow a Zipf distribution with a particular α ∈ [0, 1] parameter, we chose to use the Zipf–Mandelbrot law implemented in the zipfR R package (51) to simulate clonotype distributions. The probability density function used for simulations is given by

$$g(\pi ):=\{\begin{array}{cc} C\cdot \pi^{-\alpha -1} & 0\leq \pi \leq B\\ 0 & \text{otherwise} \end{array}$$

with two free parameters: α ∈ [0, 1] and *B* ∈ [0, 1] and a normalizing constant *C*. *B* corresponds to the probability π1 of the most frequent species (clonotype).

Seven Zipf distributions were generated with the following Zipf parameters:

A(=1/α)∈{2,3,4,5,10,20,100} and B=0.2

For each Zipf parameter combination, a list *Z_A_* is randomly generated as follows:

$$\begin{array}{l} Z_{A}=\;\{z_{A,1};z_{A,2};\ldots z_{A,\text{NA}}\},\\ \text{with}\;\forall i,z_{A,i}\in {\mathbb{R}}^{+*}\\ \;\forall i,j,\text{ if }i\leq j\text{ then }z_{A,i}\geq z_{A,j}\\ \;N_{A}=2\cdot 10^{6} \end{array}$$

*Z_A_* elements follow a Zipf distribution of *A* (= 1/α) parameter.

**Table:** 

A	2	3	4	5	10	20	100
$\sum_{i=1}^{N_{A}}z_{A,i}$	1·31·10^8^	2·10·10^7^	1·60 10^7^	1·44·10^7^	1·23·10^7^	1·16·10^7^	1·11·10^7^
***N_A_***	2·10^6^	2·10^6^	2·10^6^	2·10^6^	2·10^6^	2·10^6^	2·10^6^

C. For each *A* parameter, the 2·10^6^ *Z_A_* values were randomly assigned to the clonotype collection to obtain seven TRB clonotype repertoires.
D. To obtain the final seven datasets, each of them was rarefied using the function *rrarefy* from Vegan R package to datasets of with a size of 6·10^5^ sequences.

#### Rarefaction at Increasing Sizes

Each of the seven simulated datasets was rarefied into a series of six datasets of size *D* ∈ {500, 1,000, 5,000, 5·10^4^, 5·10^5^, 1·10^6^}. For each value of *D*, subsamples of TRB sequences were randomly produced using the *vegan:rrarefy* function (without replacement). This process was iteratively repeated 100 times with replacement. For each resulting series of subsamples, clonotype counts were calculated and used to assess the median and 95% CI values of Morisita–Horn index [MH; (52)] between them and the original dataset (representativeness) and between each other (robustness).

Subsample compositions were also compared to evaluate the level of overlap between three subsamples according to the dataset size.

For each *D*, combinations of 3 *Z_A_* dataset subsamples were randomly selected to determine the proportion of clonotypes observed once, twice or in the three subsamples. This process was performed 100 times to calculate the median and 95% CI of each result.

Since the 95% CI values obtained for MH and overlap proportion were similar to the medians, they are not indicated in the corresponding figures and tables.

## Results

### Impact of Sequencing Depth on the Representativeness of the Repertoire Diversity

**Figure 1:**
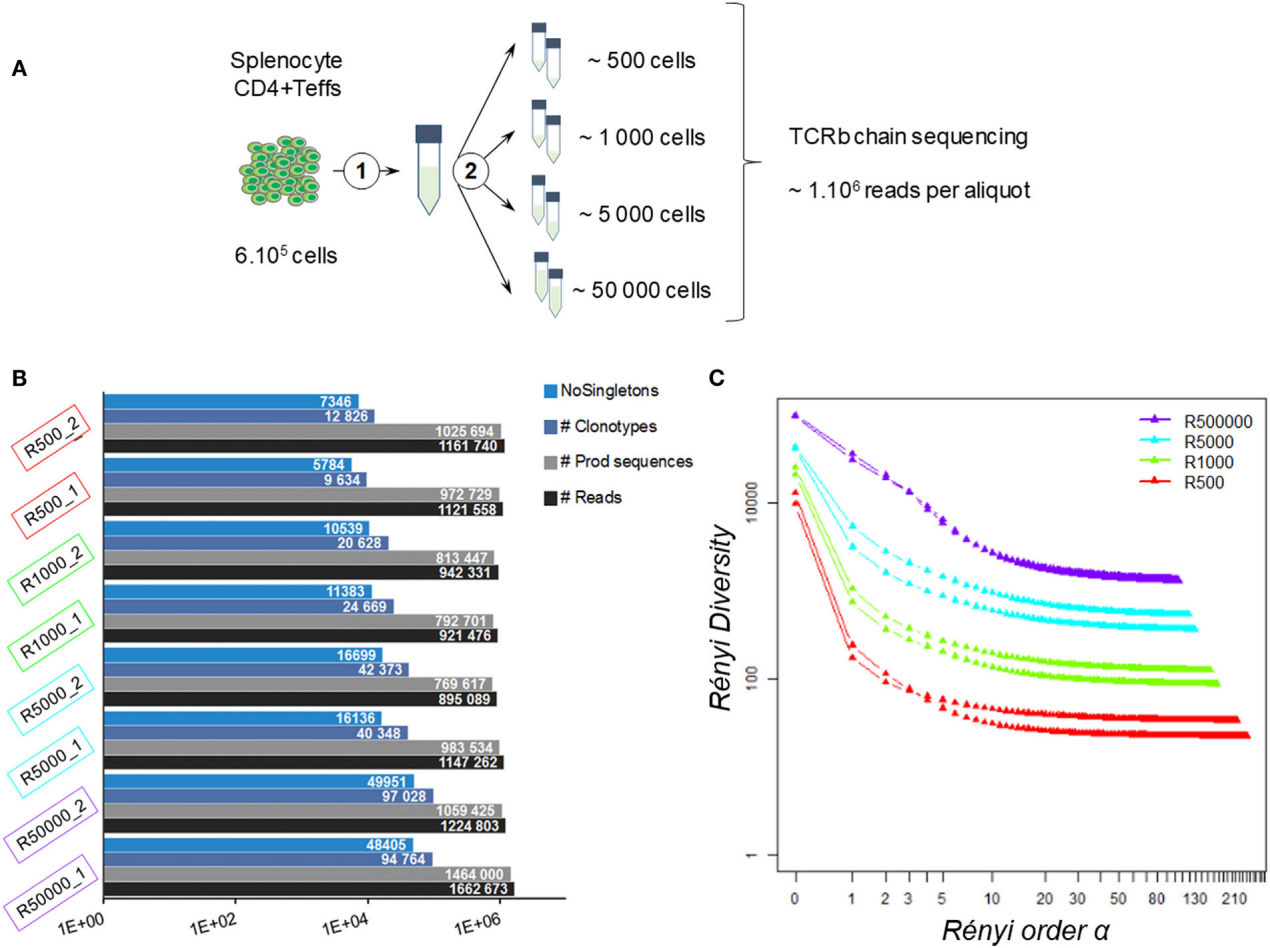
Impact of sequencing depth on measured diversity. **(A)** Experimental design: 600,000 CD4^+^Foxp3-GFP^−^ cells were sorted from female Balb/C Foxp3 splenocytes. RNA was extracted (1) and split into aliquots equivalent to the amount of mRNA of 500, 1,000, 5,000, and 50,000 cells (2). Two aliquots were produced for each amount of RNA. The eight prepared aliquots were processed for TRB chain deep sequencing. **(B)** Dataset summaries. Histograms show, for each resulting dataset, the number of reads (black), productive TRB sequences (gray), observed clonotypes (blue), and clonotypes observed more than once (NoSingletons; light blue). **(C)** Diversity profiles. For each dataset, a Rényi diversity profile was computed: diversity metrics using clonotype frequencies were calculated for increasing values of Rényi order α until stabilization of the resulting diversity. For α = 1, the Shannon entropy was computed.

With advances in HTS technologies, the minimum number of outputs in RepSeq studies is often a million sequences per sample. Besides, small samples are often studied. Thus, to determine the minimum number of sequences required for a representative repertoire, we first explored how the number of raw reads could affect the repertoire description according to the sample size. We chose to analyze a mouse sample with high diversity and used the CD4^+^Foxp3-GFP^−^ cell population (Teff) previously described as very diverse (4). 6·10^5^ Teff cells from female Balb/C Foxp3 < GFP > splenocytes were sorted. RNA was extracted from these cells and diluted in order to obtain aliquots containing the RNA amount equivalent to what would be obtained from 50,000, 5,000, 1,000, or 500 cells (Figure 1A). Two replicates per dilution were prepared. For simplicity in the text, the sample size will be defined according to the theoretical equivalent cell number for each aliquot. Sequencing was performed on RNA amplified by multiplex-PCR using a commercially available kit. We made this choice for three reasons: (1) a commercially available kit is standardized, avoiding pipetting errors in master mix preparation, (2) multiplex-PCR are template-target based, therefore we know what we are supposed to obtain in terms of V genes, and (3) the bias toward genes should be constant.

On average, 1.13 (±0.16) million reads were produced for each aliquot (Table S1 in Supplementary Material), which is in the average range of common practice (18, 44, 53). As summarized in Figure 1B, 0.99·10^6^ (±0.15·10^6^) TRB sequences were identified per aliquot regardless of the sample size. The point here is to determine whether the sample size will impact the resulting repertoire distribution.

Thus, we analyzed the diversity of the observed repertoires according to sample size. It is noteworthy that the number of unique clonotypes (i.e., unique combination of TRBV-CDR3pep-TRBJ) per sample was always higher than the number of cells per sample. This discrepancy was more marked for small size samples, with approximately 20- to 2-fold more clonotypes per sample than cells with the “500-” and “50,000-cell” samples, respectively. In each dataset, about 50% (±6%) of the clonotypes were observed once (singletons). After removing the singletons, as it is commonly done (44), this bias was reduced for the large samples, while the numbers of clonotypes remained much higher than the actual number of cells in small samples (Figure 1B). Still, overall richness remained equivalent between all sample sizes.

In order to refine the diversity assessment of these TRB repertoires, we computed their diversity profile (Figure 1C) applying Rényi entropy to the clonotype relative frequencies within each dataset. This function is used in ecological science to quantify the diversity, uncertainty, and randomness of a given system (54, 55). As the α order increases, it defines metrics spanning from (i) the species richness to (ii) the clonal dominance that progressively discards the scarcest species. The exponential of these metrics provides comparable effective numbers of species, used here to build a diversity profile. Analysis of the Rényi profiles for the eight aliquots showed that TRB repertoire diversity strongly decreases when the Rényi order α value increases. While richness was comparable between all sample sizes, diversity drops in proportion to sample size when progressively discarding scarce clonotypes to reach a plateau of clonotype counts below the initial number of cells.

### Shannon Entropy as a Threshold to Filter the Clonotypes

**Figure 2:**
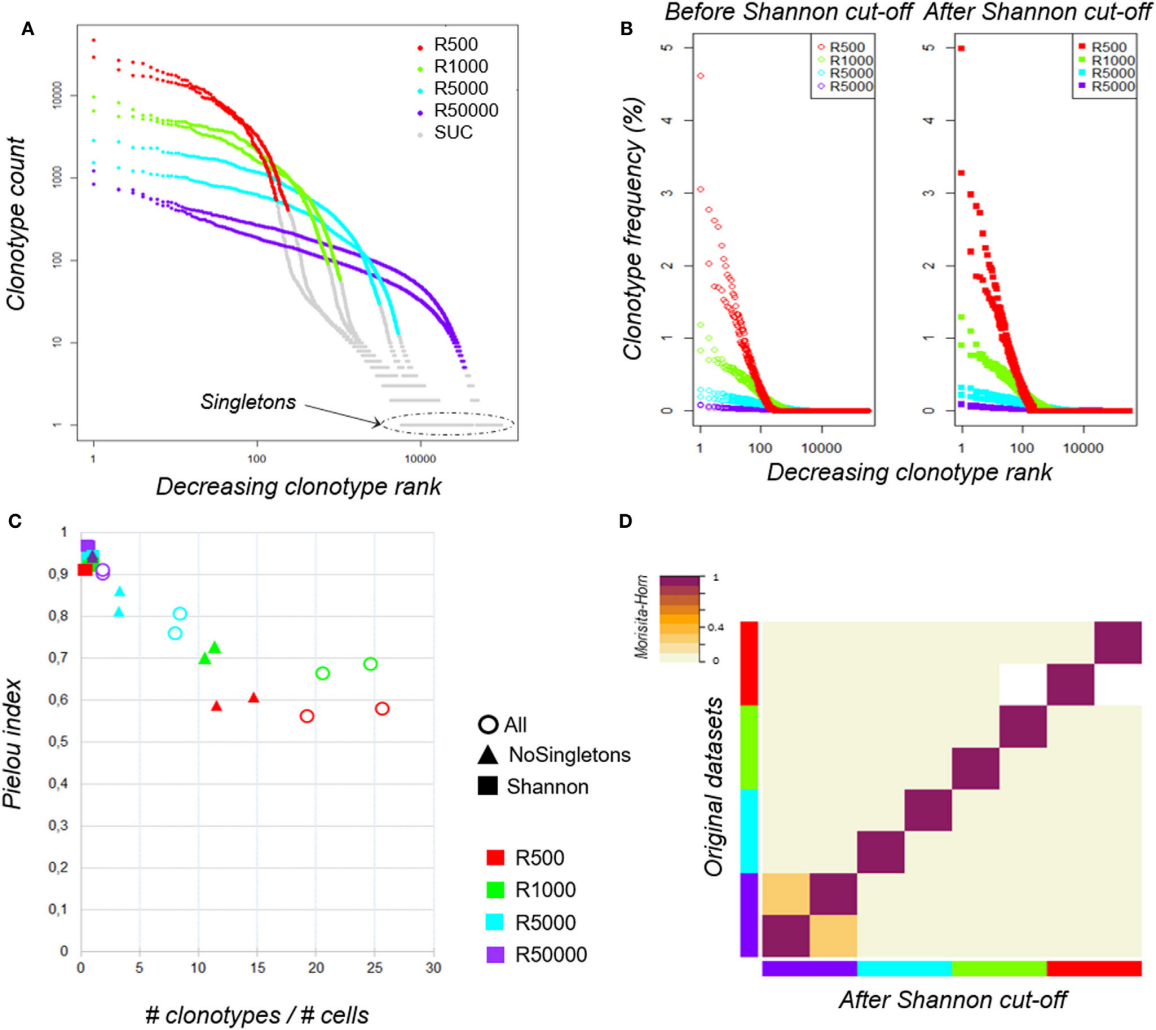
Clonotype distributions before and after data filtering. **(A)** TRB clonotype counts of the eight aliquots according to sampling size. Within each dataset, clonotypes were ranked according to their counts from the most to the least predominant (decreasing clonotype rank) and their abundance (clonotype count). Both axes are log-scaled. Plots were colored according to the sampling size: “500 cells” in red, “1,000 cells” in green, “5,000 cells” in cyan, and “50,000 cells” in purple. Clonotypes filtered out using the Shannon index (see main text) are colored in gray [scarce uninformative clonotypes (SUC)]. **(B)** TRB clonotype distributions of the eight aliquots before and after data filtering. Before (left) and after (right) filtering each dataset using the Shannon index as threshold, clonotypes were ranked from the most to the least predominant (decreasing clonotype rank) according to their relative frequencies (clonotype frequency). The X-axis is log-scaled. Distributions were colored according to the sampling size as previously. **(C)** Impact of clonotype filtering on the clonotype distribution evenness. The ratio between the number of clonotypes and the number of cells (*x*-axis) was calculated for each aliquot before (circles) and after clonotype filtering either by removing only singletons (triangles) or using the Shannon index as a threshold (squares). For each dataset, the Piélou evenness index was calculated (*y*-axis). Aliquots are identified according the sampling size as previously. **(D)** Similarity between datasets before and after Shannon filtering. The Morisita–Horn similarity index between all pairs of datasets is color-coded according to the indicated scale before (lower half-triangle) and after (upper half-triangle) Shannon filtering. Aliquots are identified according to sampling size as previously.

To avoid bias related to sample size, we normalized each dataset to 700,000 sequences, a value corresponding to the smallest sample size (Table S1 in Supplementary Material). Therefore, we randomly selected 700,000 sequences, ranked the unique clonotypes from the most to the least predominant (clonotype rank) and plotted their abundance (clonotype count) to assess their distribution (Figure 2A). It is noteworthy that, while all the aliquots come from the same sample, the clonotype distributions within each dataset are different. The smaller a sample, the higher the most predominant clonotype counts, making it difficult to apply a filtering rule based on the count values. The Rényi profiles (Figure 1C) showed that the repertoire diversity collapses at a Rényi order α of 1, which corresponds to the Shannon diversity index (56). Since the number of clonotypes assessed by the Shannon index (Table 1) correlates best with sample size (Pearson coeff = 0.966, *p* = 9.62·10^–5^ and MH = 0.877 on original clonotype number and Pearson coeff = 0.995, *p* = 2.92·10^−7^ and MH = 0.996 after clonotype number determined by Shannon index), we chose to use this metric as a threshold to discard scarce “uninformative” clonotypes (SUC) that could result from experimental noise (shown in gray in Figure 2A) and keep only “informative” ones. As shown in Figure 2B, the clonotype relative distribution within each dataset is not significantly altered by this filtering. Interestingly, as shown in Figure 2C, regardless of the initial number of cells, this transformation regularizes the values of the Piélou evenness index, a measure of clonotype evenness (57) (filled squares), which otherwise strongly decreases for unfiltered datasets when the clonotype number/cell number ratio increases, revealing that a too high sequencing depth for small samples alters clonotype distributions (Figure 2C, empty circles).

**Table 1:** Shannon diversity calculated for each dataset.

Shannon diversity	R500	R1000	R5000	R50000
#1	171	1,034	3,124	30,432
#2	238	735	5,337	35,027

To confirm that the filtering does not bias the overall repertoire diversity, we computed the Morisita–Horn (MH) similarity index between the datasets before and after filtering; the high similarity values (0.983; 0.997) shown on the matrix diagonal in Figure 2D confirm that the datasets are not altered in the process. The similarity matrix also reveals a low similarity between replicates, except for the “50,000-cell” samples, which are big enough to share rare clonotypes. Thus, high sequencing depth does not ensure good coverage of clonotype richness. This led us to question the robustness of RepSeq experiment results.

### Robustness of the TRB Repertoire Diversity Assessment by RepSeq

We sorted 3·10^6^ Teff cells from splenocytes, extracted the RNA and split it into three equivalent RNA aliquots, and then sequenced them independently at a high-depth targeting the TRB chain using the iRepertoire^®^ multiplex-PCR technology. On average, for each aliquot, 8.33 (±0.66) million reads were produced and 5.63 (±0.56) million TRB sequences were identified, among which an average of 130·10^3^ (±5·10^3^) clonotypes (Table S2 in Supplementary Material). After applying Shannon filtering, the dataset sizes were reduced to 4.7 (±0.6) million TRB sequences for a total of 44,217 (±304) clonotypes. Datasets were rarefied at an equivalent size by randomly selecting 4·10^6^ sequences for each sample.

**Figure 3:**
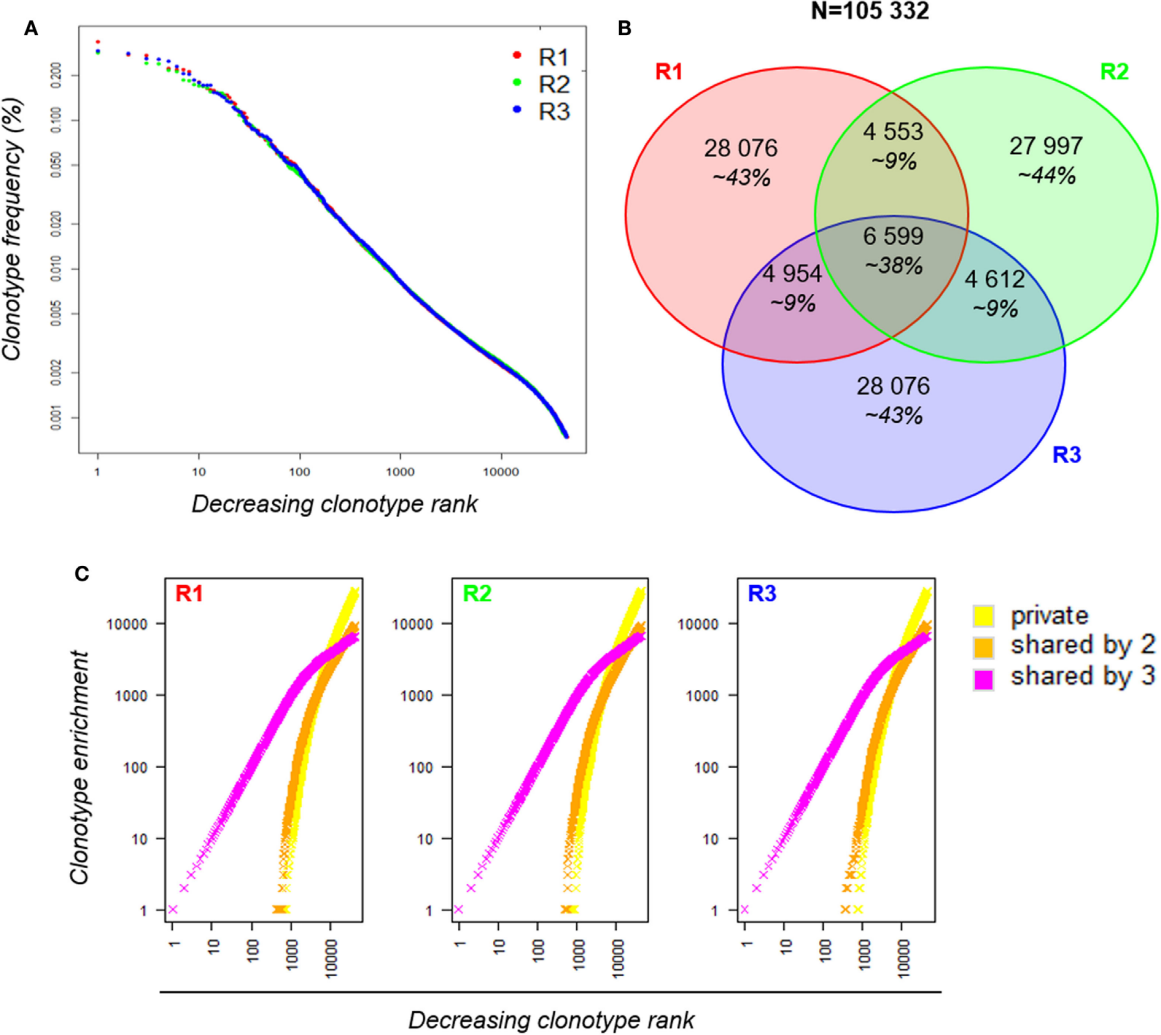
Robustness of a RepSeq experiment. **(A)** Clonotype distribution of the three replicates within each dataset. Informative clonotypes were ranked decreasingly according to their abundance and their frequency was plotted. The *x*-axis is log-scaled. **(B)** Venn diagram between the three replicates. Out of the 105,332 clonotypes observed in total, only 6,599 are shared by the three replicates; their cumulative frequency covers about 38% of each dataset. **(C)** Spectrum of unshared (yellow) and shared (by two in orange and by three in magenta) clonotypes in each replicate. Within each dataset, clonotypes were ranked according to their counts from the most to the least predominant (decreasing clonotype rank). Since clonotypes are labeled according to their sharing status, the clonotype enrichment (*y*-axis) of each sharing group is incremented (+1) when a corresponding clonotype is found in the ranked list.

We first analyzed the clonotype distributions within each dataset. The three distributions were similar between replicates (Figure 3A). However, when we compared the composition of the three TRB repertoires by clonotype overlap, it appeared that about 36% of the clonotypes observed in each dataset are shared by another replicate, with only 6,599 clonotypes common to the three replicates. Although these shared clonotypes represent only 6% of the 105,332 clonotypes identified overall, their expression accounted for approximately 38% of each repertoire (Figure 3B). We then decomposed the clonotype collection by labeling the clonotypes as private (not shared between replicates) or shared by two or three replicates. For each dataset, clonotypes were sorted from the most to the least abundant and enrichment curves were built for each category according to the sharing status of each clonotype (Figure 3C). The resulting clonotype spectrum revealed that the most predominant clonotypes are shared by the three replicates, while the private clonotypes, which are the more numerous, are enriched for scarce clonotypes, therefore reducing the similarity between technical replicates. These results demonstrate that although the sampling of a large and polyclonal cell population has no impact on the observed clonotype distribution, the repertoire composition is affected: even if the most predominant clonotypes are always captured, a major proportion of the clonotypes observed with a single sequencing are private scarce ones. This observation confirms that the more abundant a clonotype, the more likely it is to be observed by sequencing. However, most rare clonotypes will remain unseen with a single sequencing run.

### Computational Assessment of the Impact of Sequencing Depth on Observed Diversity

In order to assess the representativeness of the diversity observed when analyzing a clonotype repertoire by RepSeq, it would be necessary to know *a priori* its full diversity and distribution, which is not achievable with a classic experimental approach inherently subject to sampling bias.

Several studies have demonstrated that immune repertoires follow a Zipf-like distribution (58–62), which translates a relation between rank order and frequency of occurrence: the frequency *f* of a particular observation is inversely proportional to its rank *r* (63) with:

$$f\left(r\right)\propto \frac{1}{r^{\alpha }}$$

for Zipf-α parameter ≈ 1 (64).

In addition, the lower the Zipf-α parameter of a distribution, the more evenly represented the clonotypes involved (59). We applied this observation to build clonotype distributions of a fixed size and known diversity to simulate the sampling effect occurring during a RepSeq experiment.

Seven Zipf distributions of 6·10^5^ sequences each were simulated with a parameter A = 1/Zipf-α ranging from 2 to 100. These distributions were then assigned to a list of clonotypes randomly generated using the tcR package (65), leading to seven TRB clonotype repertoires of known diversity.

**Figure 4:**
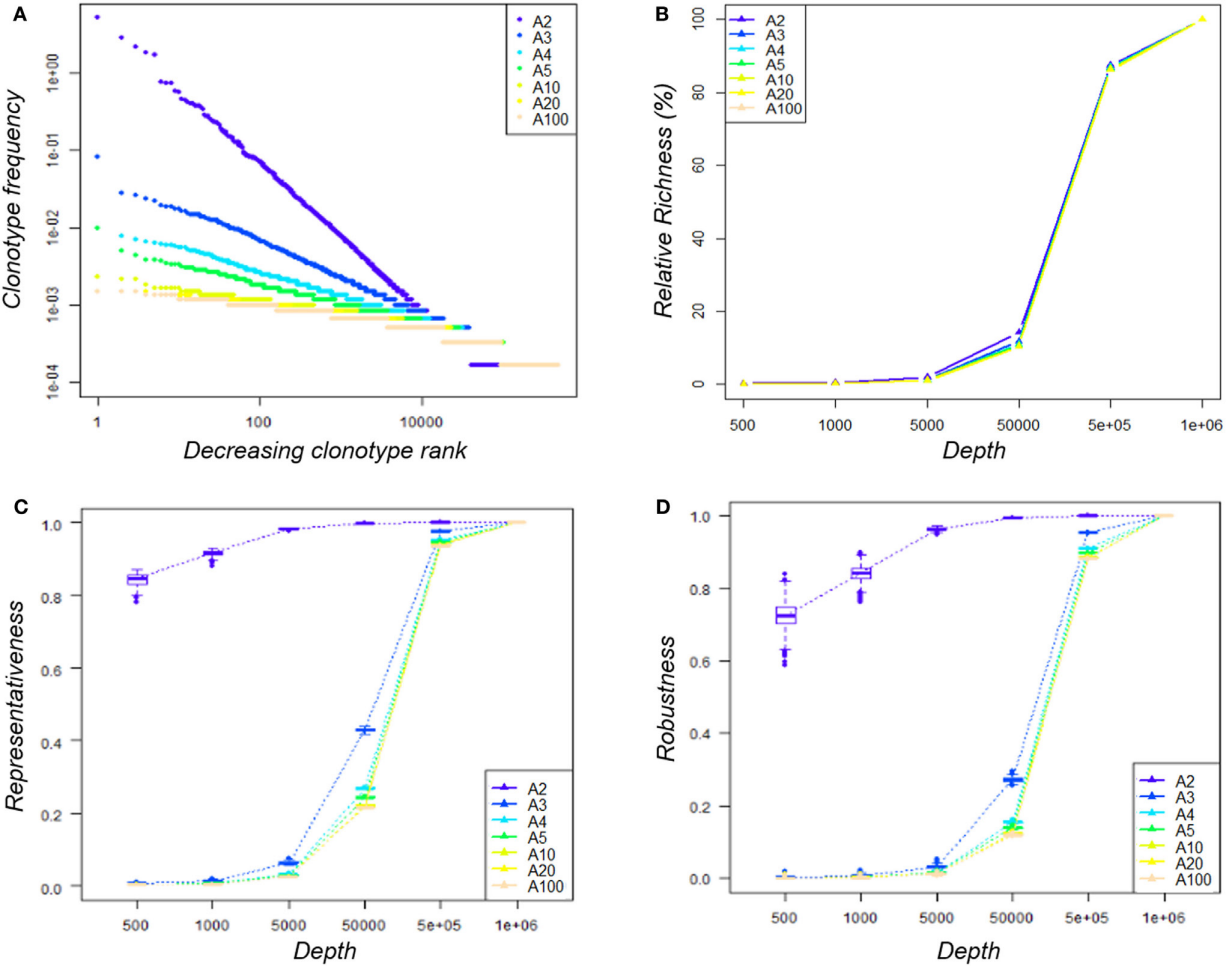
Impact of sequencing depth on the observed diversity. **(A)** Clonotype distribution within the seven simulated datasets—within each *A*-dataset, clonotypes were ranked decreasingly according to their abundance and their frequency was plotted. Both axes are log-scaled. Distributions are colored according to the *A* parameter used to simulate it. **(B)** Impact of sequencing depth on the observed clonotype richness—for a given *A*-dataset, clonotype richness was measured within the 100 subsamples produced for each depth and divided by that of the original dataset. The median value by depth is represented for each condition. The 95% CI was calculated but cannot be seen since it merged with the median value. **(C)** Representativeness of the sequencing—the Morisita–Horn similarity index was calculated between each subsample and its original dataset. Boxplots across the 100 subsamples of a given depth are color-coded according to the *A* condition. **(D)** Reproducibility of the sequencing—for each *A*-dataset, the Morisita–-Horn similarity index was calculated between paired subsamples of a given depth. Boxplots across the 100 subsamples of a given depth are color-coded according to the *A* condition.

As observed in Figure 4A, the distribution slope varies according to the depth of sequencing of the clonotypes. For example, for the distribution simulated with *A* = 2 (A2), the resulting distribution is skewed in a way that clonotype counts range from 1 to 31,109, whereas when *A* = 100 (A100), clonotype counts do not exceed 9. These different distributions lead to datasets of varying richness, as summarized in Table 2.

**Table 2:** Summary of the simulated Zipf distributions.

A	2	3	4	5	10	20	100
$\sum_{i=1}^{N_{A}}z_{A,i}$	6E + 05	6E + 05	6E + 05	6E + 05	6E + 05	6E + 05	6E + 05
***N_A_***	155,495	394,784	435,528	450,625	469,974	476,829	480,919

For each of our seven “known” repertoire distributions, we generated 100 subsamples at 6 sample sizes (from 500 to 1·10^6^ sequences) reflecting several levels of sequencing depth. The clonotype richness observed within each subsample increased according to the depth, as expected (Figure 4B). We used the MH similarity index to assess (i) representativeness (Figure 4C) by comparing the diversity captured for each subsample with the original repertoire diversity and (ii) reproducibility (Figure 4D) for the 100 subsamples for a given depth. When comparing the seven distributions at a given sequencing depth (5·10^4^ sequences, representing 8% of the original repertoire), the representativeness of the diversity between distributions was different (Figure 4C), yet with similar relative richness values. For the “A2” condition, the similarity index between this subsample and the original repertoire was above 0.8, while it varied from 0.2 to 0.5 for the other conditions (Figure 4C). A dataset of 5·10^5^ sequences (80% of the original repertoire size) is needed to reach a 0.9 similarity for the latter. However, a suitable representativeness does not ensure good reproducibility of the observations. With 500 or 1,000 sequences, even if the diversity observed for the “A2” condition is quite representative (MH ~ 0.8), the high variability between the subsamples implies a low reproducibility and thus an inability to observe exhaustively all the clonotypes (Figure 4D).

**Figure 5:**
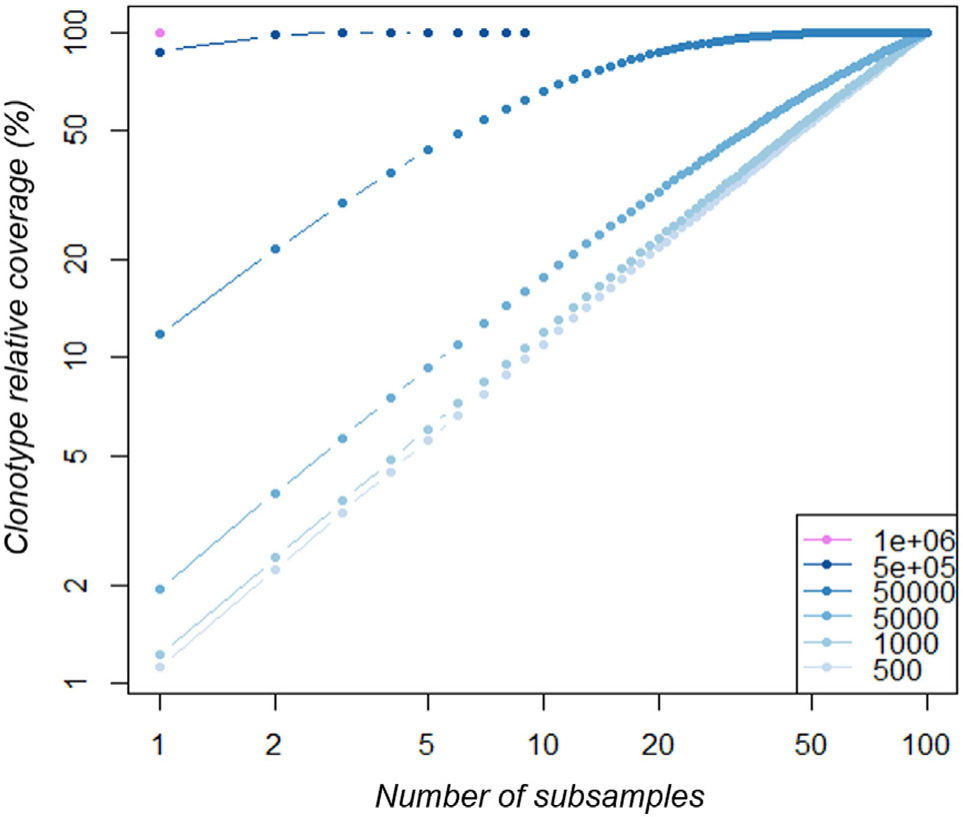
Clonotype coverage of A3-dataset richness increases with multiple subsamples. The A3-datasets were subsampled at increasing depth (from 500 to 1·10^6^ sequences as indicated in the legend from light to dark blue). For each depth, 100 subsamples were produced. Within each subsample series, an increasing number of subsamples (*x*-axis) were randomly selected and their cumulative clonotype richness was calculated relative to the original dataset richness (clonotype richness coverage).

We sought to identify which simulated distribution would be the most representative of our experimental datasets. To this end, we compared the slope at the steepest descent point of each simulated distribution with those of all the experimental data analyzed in this study. The experimental distribution slopes are most comparable with the “A3” and “A5” distributions, with the exception of that of the R500_2 sample (Table S3 in Supplementary Material). Thus, we chose the “A3” distribution dataset as the most representative. In order to understand the low overlap observed between experimental replicates in Figure 3B, for each size we compared the “A3” simulated subsamples to determine the proportion of clonotypes shared by three independent subsamples, as performed experimentally in Figure 3. As summarized in Table 3, the proportion of private and shared clonotypes varies according to the coverage of the initial repertoire stretch. For subsamples with sizes representing less than 1% of that of the initial dataset, almost all the clonotypes observed are private (only captured in one subsample). For the “5·10^4^ sequence” subsamples, the size of which represents 8% of the original repertoire size, 16% of the clonotypes observed are captured at least twice. These proportions correspond to the observations we made in Figure 3 between the three experimental replicates. Finally, using subsamples of size close (80%) to that of the original, 95% of the observed clonotypes are shared by at least two replicates. In addition, as represented in Figure 5, at this depth, while one sample only captures about 12% of the overall existing clonotypes, three replicates cover a third of the overall richness. These observations suggest that multiple sequencing experiments can ensure greater clonotype exhaustiveness than a unique very deep sequencing.

**Table 3:** Sharing proportion between three replicates.

Median proportion of clonotypes observed	Dataset sizes					
	5·10^2^	1·10^3^	5·10^3^	5·10^4^	5·10^5^	1·10^6^
Private	99.7	99.4	97.5	83.7	5.2	–
Shared by two	0.3	0.6	2.4	14.3	27.2	–
Shared by three	–	–	0.1	2	67.6	100
Total number across three replicates	1,493	2,973	14,456	117,634	393,434	394,784

## Discussion

RepSeq offers new opportunities to identify biomarkers of health or disease by monitoring adaptive immune cell diversity at unprecedented high resolution. Continuing improvements in molecular biology protocols and sequencing technologies are increasing the accuracy of clonotype detection (66). Still, clear evaluation of the reproducibility and representability of the observed diversity is missing. This is particularly true when considering bulk sequencing on small size samples such as small cell subsets or cells from biopsies, though of utmost interest when studying TCR repertoires. Although over-sequencing has been recommended to ensure the identification of rare clonotypes (53), it does increase the risk of generating uninformative, possibly artifactual clonotypes such as duplicate reads and chimeric reads (67). Indeed, when sequencing samples of varying sizes at a commonly used depth, we found that small datasets contained 20 times more clonotypes than would be expected regarding the sample size. This figure decreases when the starting material is increased, demonstrating that over-sequencing small samples dramatically generates noise that cannot be corrected by removing only singletons. Although the relationship between sample size and sequencing depth that we used may appear extreme, it can commonly occur when studying small cell subsets involved in immunological processes. These observations demonstrate the drawbacks of discarding clonotypes based only on their counts and the need for objective approaches in order to assess the actual richness of a repertoire effectively. Single-cell sequencing technologies are an alternative to accurate study of the repertoire of small cell subsets and therefore will surely not require the use of Shannon filtering, because the number of expected unique TR sequences will be at most two per single cell. However, currently the number of required cells is still regularly higher than actually recovered in particularly low-input samples.

Here, we provide a bioinformatics approach to assess accurately the number of unique clonotypes in a large and complex cell population, even when over-sequenced. When analyzing the diversity profiles of repertoires from subsamples of varying sizes of a unique starting sample, we identified Shannon entropy as a reliable threshold to eliminate clonotypes arising from technical noise (SUC) and to focus on informative TR clonotypes (Figures 1C and 2A). This filtering strategy has no impact on the overall clonotype distribution (Figure 2B). Importantly, this approach was validated on subsamples originating from a single starting sample. Therefore, the representability of the smallest subsample was questioned. While the distribution evenness was sample size-dependent when considering all the reads, filtering by the Shannon entropy index removed this variability between replicates (Figure 2C). This proposed strategy therefore offers an accurate assessment of clonotype identification and representability, even in extreme situations. We applied our method to data produced following multiplex-PCR amplification on bulk polyclonal CD4^+^ T cells, for which the targeted genes and bias should be constant from one experiment to another. Although the number of uninformative clonotypes should be assessed when analyzing datasets prepared by different molecular methods, we believe that the Shannon index should reflect the true diversity by excluding uninformative clonotypes. Once single-cell sequencing becomes standardized and applicable to a range of very small to very large sample sizes, such correction metrics may not be necessary anymore.

Our results strongly suggest that sequencing depth must be adapted to the initial cell amount. We show that “50,000-cell” replicates are closer to each other than lower input pairs of samples (Figure 2D). This observation emphasizes the need to adapt the sample size to the population of interest. All aliquots analyzed here were obtained from a rich and polyclonal cell population. In order to be reliable, a sample needs to be large enough to ensure that most of the clones are represented. Here, about 20% of the clonotypes observed in the two replicates (6,766 out of 30,422 and 35,020 clonotypes) are shared.

Altogether these results show how complex defining a RepSeq strategy can be in guaranteeing the representativeness of the repertoire diversity. If sequencing depth is not adapted to the population size, it can negatively affect the resulting observed diversity, in particular if data are not properly analyzed. This is particularly crucial since the clonality of a population is rarely known before its sequencing, leading to misinterpretation of the results. Since the sequencing depth used was much higher than the size of the samples we analyzed, one would expect good, if not exhaustive, coverage of the overall clonotypes. Conversely, we show that this is by no means the case, with only part of clonotypes being observed with confidence. These observations led us to question the robustness of the results of RepSeq experiments.

Multiple sequencing of the same sample revealed very low overlap between technical replicates, even after filtering out uninformative TR clonotypes, and merely captures the most frequent clonotypes. Rare clonotypes were at best shared by two replicates. As already suggested by Greiff et al. (44), our results are in favor of multiple sequencing when considering very diverse samples. This can be explained by the experimental sampling enforced by the different RepSeq steps (from RNA amplification to library sequencing). In order to validate these experimental observations and propose guidelines for RepSeq studies, we simulated different repertoire distributions and found that the representativeness of a very evenly distributed repertoire, which could be likened to a polyclonal repertoire, is more sensitive to the sequencing depth. The number of sequences produced (by multiple sequencing) needs to be equivalent to the population size to ensure a good assessment of the original diversity (Figure 4C). This is particularly true for small samples for which too deep a sequencing can favor the erroneous sequences possibly generated during library preparation (68) and thereby introduce experimental noise.

Altogether, we provide here a method that accurately discards uninformative clonotypes for small and large samples based on the application of Shannon diversity index threshold filtering, as well as guidelines for RepSeq experimental design. In addition, we show how computational simulation of diversity can improve adaptive repertoire analysis assessment where controlled reference repertoires with known actual diversity can be modeled and subject to experimental design and annotation tool flaws. We believe these will be useful in ensuring better RepSeq analyses when looking at rare or unknown cell populations participating in pathophysiological processes and will facilitate the discovery of HTS-based biomarkers.

## Ethics Statement

This study was carried out in accordance with the recommendations of the “European legislation on animal care, housing, and scientific experimentation under the agreement number A751315.” The protocol was approved by the “local animal ethics committee.”

## Authors Note

The RNA sequences presented in this study have been submitted to Sequence Read Archive (SRA; https://www.ncbi.nlm.nih.gov/sra) as Bioproject PRJNA408306, under accession numbers SRR6068973, SRR6068972, and SRR6068975 (Biosample SAMN07682929) and SRR6068974, SRR6068969, SRR6068968, SRR6068971, SRR6068970, SRR6068967, SRR6068966, and SRR6068976 (Biosample SAMN07682930).

## Author Contributions

WC performed all the bioinformatics analyses. AG-T and L-MF prepared the samples. WC, EM-F, AS, and DK conceived the studies, designed the experiments, and analyzed the results. WC, EM-F, AS, and DK wrote the first draft of the manuscript, with input from all authors. DK initiated and obtained funding for the study. EM-F and AS contributed equally to the work.

## Conflict of Interest Statement

The authors declare that the research was conducted in the absence of any commercial or financial relationships that could be construed as a potential conflict of interest.
