# Supplementary material for: RepSeq Data Representativeness and Robustness Assessment by Shannon Entropy
Source: Front Immunol. 2018 May 15;9:1038. doi: 10.3389/fimmu.2018.01038 (PMC5962720; doi:10.3389/fimmu.2018.01038)
Supplement: Supplementary file 1 [file Table_1.docx]

Supplemental Table I: Descriptive statistics of HTS datasets in Figure 1

| Step | 1 | 2 | 3 | 4 | 5 | | | 6 | 7a | 7a' | 7b | |
| --- | --- | --- | --- | --- | --- | --- | --- | --- | --- | --- | --- | --- |
| **ID** | **Raw reads** | **TRB sequences before processing** | **Productive TRB sequences** | **Productive TRB sequences error corrected** | **V** | **J** | **V-J** | **Unique clonotype** | **Singleton** | **Unique clonotype w/o singletons** | **Unique clonotype after Shannon filtering** | |
| **R500_1** | 1121558 | 1027510 | 972729 | 907466 | 21 | 13 | 122 | 9634 | 3850 | 5784 | 171 | |
| **R500_2** | 1161740 | 1069317 | 1025694 | 948734 | 21 | 13 | 153 | 12826 | 5480 | 7346 | 238 | |
| **R1000_1** | 921476 | 834170 | 792701 | 729662 | 21 | 13 | 180 | 24669 | 13286 | 11383 | 1034 | |
| **R1000_2** | 942331 | 856799 | 813447 | 747211 | 21 | 13 | 173 | 20628 | 10089 | 10539 | 735 | |
| **R5000_1** | 1147262 | 1037905 | 983534 | 902401 | 21 | 13 | 208 | 40348 | 24212 | 16136 | 3124 | |
| **R5000_2** | 895089 | 808524 | 769617 | 703387 | 21 | 13 | 213 | 42373 | 25674 | 16699 | 5337 | |
| **R50000_1** | 1662673 | 1533677 | 1464000 | 1348943 | 21 | 13 | 239 | 94764 | 46359 | 48405 | 30432 | |
| **R50000_2** | 1224803 | 1116768 | 1059425 | 968744 | 21 | 13 | 235 | 97028 | 47077 | 49951 | 35027 | |
| For each sample (ID), the numbers correspond to the actual number of the sequences identified at each step. | | | | | | | | | | | |  |
| *The pipeline includes the following steps: 1: Counting of raw reads; 2: Annotation of raw reads with clonotypeR and counting the TRB sequences as sequences where a TRBV, a CDR3 and a TRBJ have been identified. No error correction performed at this step; 3: TRB sequences are cleaned from non-productive and ambiguous sequences. No error correction performed at this step; 4: TRB productive sequences are corrected for PCR/sequencing errors (see material and method); 5: V, J and VJ combination are counted on unique clonotypes; 6: Counting of unique clonotypes; 7: Filtering steps are performed on unique clonotypes (7a/a' or 7b). Figure 1B represents values from step 1 (# Reads), step 3 (“# Prod Sequences” bar), step 6 (“# Clonotypes” bar) and step 7a’ (“NoSingletons” bar). Unique clonotype count after Shannon filtering (step 7b) are represented in Figure 1 C for Rényi order α = 1. | | | | | | | | | | | | |
