# Supplementary material for: RepSeq Data Representativeness and Robustness Assessment by Shannon Entropy
Source: Front Immunol. 2018 May 15;9:1038. doi: 10.3389/fimmu.2018.01038 (PMC5962720; doi:10.3389/fimmu.2018.01038)
Supplement: Supplementary file 2 [file Table_2.docx]

Supplemental Table II: Descriptive statistics of HTS datasets used in Figure 3

| **ID** | **Reads** | **TRB sequences before processing** | **V** | **J** | **V-J** | **Clonotypes** | **Singletons** | **Shannon diversity** | **TRB sequences after processing** |
| --- | --- | --- | --- | --- | --- | --- | --- | --- | --- |
| **R1** | 7 345 806 | 6 836 292 | 23 | 14 | 258 | 136 101 | 14 255 | 44 647 | 5 427 779 |
| **R2** | 9 178 548 | 5 732 884 | 23 | 14 | 260 | 128 123 | 13 791 | 43 761 | 4 567 202 |
| **R3** | 8 469 835 | 5 290 615 | 23 | 14 | 259 | 127 403 | 12 749 | 44 241 | 4 193 317 |
