# Supplementary material for: RepSeq Data Representativeness and Robustness Assessment by Shannon Entropy
Source: Front Immunol. 2018 May 15;9:1038. doi: 10.3389/fimmu.2018.01038 (PMC5962720; doi:10.3389/fimmu.2018.01038)
Supplement: Supplementary file 3 [file Table_3.docx]

Supplemental Table III: Slope coefficients at point of inflection for simulated (A) and experimental (B & C) HTS datasets

| **A** | **A=2^a^** | **A=3** | **A=4** | **A=5** | **A=10** | **A=20** | **A=100** |
| --- | --- | --- | --- | --- | --- | --- | --- |
| **Slope** | -2,31E+00 | -5,53E-02 | -2,17E-03 | -4,83E-03 | -3,33E-04 | -1,67E-04 | -1,67E-04 |

^a^ “A” values for the 1/Zipf-α parameter used for simulated datasets in Figure 4.

| **B** | **R50000_1** ^b^ | **R50000_2** | **R5000_1** | **R5000_2** | **R1000_1** | **R1000_2** | **R500_1** | **R500_2** |
| --- | --- | --- | --- | --- | --- | --- | --- | --- |
| **slope** | -3,69E-02 | -1,03E-02 | -4,59E-02 | -2,63E-02 | -1,39E-01 | -1,94E-01 | -2,92E-01 | -2,81E+00 |

^b^ HTS datasets generated for use in Figure 1.

| **C** | **R1** ^c^ | **R2** | **R3** |
| --- | --- | --- | --- |
| **slope** | -6,35E-02 | -3,85E-02 | -2,33E-02 |

^c^ HTS datasets generated for use in Figure 3.
